# The wellbeing pandemic: Outline of a contested terrain and a proposed research agenda

**DOI:** 10.3389/fsoc.2022.950557

**Published:** 2022-12-06

**Authors:** Steven J. Jackson, Michael P. Sam, Marcelle C. Dawson, Daniel Porter

**Affiliations:** ^1^School of Physical Education, Sport and Exercise Sciences, University of Otago, Dunedin, New Zealand; ^2^Department of Sociology, Gender Studies and Criminology, University of Otago, Dunedin, New Zealand; ^3^Centre for Social Change, University of Johannesburg, Johannesburg, South Africa

**Keywords:** wellbeing, pandemic, contested terrain, wicked problem, wellbeing washing, alternative futures

## Abstract

Wellbeing has emerged as a central, if not defining, feature of contemporary social life. Yet, despite its global significance spanning the political, social and economic spectrum, there is a remarkable lack of agreement regarding the conceptualization, definition or operationalisation of wellbeing nor any clear evidence of its success as an instrument of policy. This essay explores the contested terrain of wellbeing by examining the concept in relation to emerging politics, complexities and contradictions. More specifically, the essay: (1) briefly describes the historical origins and development of wellbeing; (2) discusses how it has been reconceptualised within the context of neoliberalism; and, (3) outlines a research agenda offering three ways to investigate wellbeing including: (a) as a wicked problem; (b) as part of the process of “wellbeing washing” within state and other institutional structures and policies; and, (c) in relation to alternative futures, which might encourage us to reimagine or jettison the term altogether.

## Introduction

Although its origins can be traced to antiquity, wellbeing has emerged as a central, if not defining, feature of contemporary social life. In response to rising global social inequalities, new conceptualisations of wellbeing have emerged that have shifted the focus from primarily economic measures, such as Gross Domestic Product (GDP) and standard of living, to allegedly more holistic and progressive measures associated with quality of life. Arguably, the accelerated, overwhelmingly positive, and largely unquestioned, trajectory of wellbeing has been so strong that it exhibits elements of a halo effect. Viewed positively, wellbeing has come to be regarded as a panacea for many societal ills spanning health, inequality and even the environment. However, viewed through a more critical lens there are increasing concerns that the politicization, commodification and exploitation of wellbeing has led to it becoming a casualty of modernity (Carlisle et al., 2009), contributing to widespread cultural anxiety (White, 2017) and impacting on individual and collective health and happiness. In this perspectives essay, we assert that if wellbeing were a disease, its global transmission combined with its complex mutations of meaning, usage, and appropriation might jocularly be said to constitute a *pandemic*. The social and geographic footprint of the concept is staggering and manifests within popular discourse and myriad institutions, ranging from the World Health Organization (WHO), United Nations (UN), World Bank, Organization for Economic Co-operation and Development (OECD), national governments and their various state sectors, workplaces, and consumer lifestyle products and services (Cederström and Spicer, 2015). Yet, despite its global significance spanning the political, social and economic spectrum, there is a remarkable lack of agreement regarding the definition, conceptualization, or operationalisation of wellbeing, nor any clear, longitudinal evidence of its success as an instrument of policy.

## The challenge of defining and conceptualizing wellbeing

To begin, let us consider the challenge of defining and conceptualizing wellbeing. Pollard and Lee (2003, p. 60), for example, note that wellbeing is: “a complex, multi-faceted construct that has continued to elude researchers' attempts to define and measure.” Likewise, Thomas (2009, p. 11) argues that wellbeing is, “intangible, difficult to define and even harder to measure”. One key area of confusion is the conflation of “wellbeing” with concepts as diverse as happiness, quality of life, life satisfaction, flourishing, and wellness. As Forgeard et al. (2011, p. 81) suggest, “[t]he question of how wellbeing should be defined (or spelt) still remains largely unresolved, which, has given rise to blurred and overly broad definitions”. Finally, White and Blackmore (2015, p. 4) advise that: “The ubiquity of references to wellbeing and the diffusion of meanings they bear means any attempt to summarize the field must inspire some trepidation”. In short, for a concept that is at the center of contemporary social life, there is very little clarity about what it means and/or how it should be measured.

Ultimately, what we do know is that by virtue of its intersection with politics, economics, health, education, and consumer lifestyles–wellbeing is now a key concept within an ever-expanding network of discourses and policies linked to power, resources and responsibility. This essay explores the contested terrain of wellbeing by examining the concept in relation to emerging politics, complexities and contradictions. More specifically, the essay: (1) briefly describes the historical origins and development of wellbeing; (2) discusses how it has been reconceptualised within the context of neoliberalism and, thus, redefined as an individual responsibility; and, (3) outlines a proposed research agenda offering three ways to investigate wellbeing including: (a) as a wicked problem; (b) as part of the process of “wellbeing washing” within supranational, state and corporate institutional structures and policies; and, (c) in relation to alternative futures, which might encourage us to reimagine or jettison the term altogether.

## A (very) brief history of wellbeing

Wellbeing has a long history and embodies diverse meanings ranging from quality of life, happiness, flourishing health, and extending to morality and mindfulness (Dodge et al., 2012; Seligman, 2012; Davies, 2015; Smith and Reid, 2017; Leary, 2019). The basic idea of wellbeing can be traced to Aristotle (Dalingwater et al., 2019) but its dominant contemporary conceptualisations are rooted in Western logic and philosophy. For example, in 1776, America's Declaration of Independence cited “the pursuit of happiness” as an “unalienable right” of citizens. In that same year, Jeremy Bentham identified happiness as a social measure to promote “utility” or the “greatest happiness of the greatest number” (Bentham, 1776). Like Bentham, English philosopher, political economist and Member of Parliament, John Stuart Mill (1806–1873) sought to use utilitarian principles to inform both law and social policy. However, he held different views with respect to how happiness and wellbeing should be cultivated and promoted. Mill's liberalism suggested that “the free development of individuality is one of the leading essentials of wellbeing” (Mill, 1859). This articulation of utilitarianism with liberalism facilitated the emergence of *homo economicus*, a model of human behavior with significant implications for social structure (see discussion of “neoliberalism” below).

Today, most conceptualisations of wellbeing are framed along two main lines. The first—subjective wellbeing—emphasizes a comprehensive, multidimensional measure of an individual's mental, physical and spiritual health (Diener et al., 2018). This perspective is perhaps best reflected in the World Health Organization's Well-being Index (World Health Organization, 1998). In 1998, the World Health Organization developed the WHO-5 Index to measure the subjective (affective and hedonistic) wellbeing of people aged 9 years or older. The index contains five statements covering states of cheerfulness, calmness, vigor, restfulness and fulfillment.^1^ The WHO-5 Index is amongst the most utilized questionnaires for assessing subjective psychological wellbeing, has been translated into more than 30 languages, and has been widely used in research studies all over the world. Moreover, according to a systematic review of literature “The scale has adequate validity both as a screening tool for depression and as an outcome measure in clinical trials and has been applied successfully as a generic scale for well-being across a wide range of study fields” (Topp et al., 2015; p. 174). However, the WHO-5 Index is not without its critics. Kusier and Folker (2020), note that the index “exhibits a range of psychometric compromises in the translation of philosophical theory into practice” (p. 338). For example, the WHO-5 index focuses on the frequency of the positive aspects of emotions but has a blind spot with respect to negative emotions and the intensity and duration of these emotions (Kusier and Folker, 2020). In addition, we assert that attempting to distill the complexity of the concept into five basic questions in order to quantify and operationalise it is overly simplistic. Furthermore, it highlights the entrenched individualization of wellbeing, that is, the tendency to rationalize, measure, and articulate wellbeing predominantly in relation to the psychological state of individuals. The overall influence of the WHO-5 index should not be underestimated given that governments, corporations, health organizations, schools, universities and a range of other sectors have not only embraced but implemented it. Such is the current hegemony of the wellbeing agenda (Harvey, 2005) that individuals daring to question its validity are often marginalized and branded as malcontents or labeled as persons suffering from ill-being (Cederström and Spicer, 2015).

The second conceptualization—objective wellbeing—captures the aggregate dimensions of the concept and is understood as an alternative to Gross Domestic Product (GDP) and a measure (ranking) of a nation's overall prosperity (Western and Tomaszewski, 2016). Consider, the OECD Framework for Measuring Well-Being and Progress which was developed on the basis of the recommendations of the 2009 Commission on the Measurement of Economic Performance and Social Progress (to which the OECD contributed significantly). This framework is built around three distinct components: current well-being, inequalities in well-being outcomes, and resources for future well-being (www.oecd.org). Like the WHO-5 index, the OECD Well-being Framework has informed a wide range of scholarly analyses across a range of academic disciplines and has been used as the basis of policy development internationally in relation to many social sectors including the economy, health and education. However, like the WHO-5 index, the OECD Well-being Framework also exhibits a range of limitations, including a lack of consensus regarding validity and the components and determinants of wellbeing (Cavalletti and Corsi, 2018). Beyond this, both the WHO-5 and OECD approaches to wellbeing have been scrutinized because (1) both operate from the basic assumption that we can objectively measure wellbeing (Alexandrova, 2018) and, furthermore, that the compulsive drive to achieve international conceptual coherence and consensus is leading, perhaps unintentionally, to the obfuscation of critical differences (Auld and Morris, 2019); (2) related to the previous point is the fact both the WHO and OECD conceptualisations and measurements of wellbeing are largely based on Western traditions thus marginalizing alternative, perspectives including, for example, Asian, Indigenous and other cultural orientations (Tiberius, 2004; Tov and Diener, 2009; Jorm and Ryan, 2014; Rappleye et al., 2020); and, (3) both operate within a wider set of power relations linked to nation-states, the United Nations, World Bank, IMF and a range of other political-economic actors that influence international affairs.

Although these two frameworks of wellbeing appear distinct, they are interrelated at least to the extent that they remain rooted within both positivist and neoliberal paradigms. On one hand, the introduction of wellbeing as a new and purportedly more progressive measure of national economic and social outcomes signals societal change, optimism and hope. On the other hand, the translation of state level policies and associated performance measures, tends to focus on *individual* wellbeing. Consequently, being “well” is defined as one who is: healthy, productive, efficient, resilient, obedient and loyal—characteristics that ensure compliance, reduce costs and increase economic growth (Cederström and Spicer, 2015). Thus, contemporary wellbeing remains embedded within a context underscored by a combination of utilitarianism and neoliberalism (Vallelly, 2021) and continues to operate within the logic of the new spirit of capitalism, a rejuvenated system of accumulation reframed in terms of liberation, security and fairness (Boltanski and Chiapello, 2005, 2007). Next, we elaborate on the emergence of wellbeing as an instrument of neoliberalism and its implications for society before proposing ideas for a new research agenda.

## Neoliberalism and wellbeing

According to the Global Wellness Institute^2^, the “wellness economy” was estimated at $US4.9 trillion in 2019 with a prediction that it could reach $US7 trillion by 2025 (Global Wellness Institute, 2021). These trends are arguably part of a wider process of market liberalization that has operated, albeit in varying manifestations and degrees, as a dominant socio-economic paradigm since the 1980s. Inasmuch as the neoliberal agenda has become endemic (Giroux, 2008; Chapman, 2016; Springer et al., 2016), wellbeing now carries a “(neo)liberal inflection” (Rappleye and Komatsu, 2020) with an emphasis on the articulation of state and individual interests (Harvey, 2005). Capturing the tension between state (objective) and individual (subjective) frameworks of wellbeing, White and Blackmore (2015) observe that:

Politically, wellbeing gives voice to desires for an alternative, a new moral economy, a counterweight to the excesses of capitalism…Its claim to put people's own perspectives at the heart of policy-making promises more democratic processes, or even empowerment (pp. 4-5)….But it may also intensify self-monitoring, with greater pressure to produce and perform happiness or [subjective] wellbeing as a marker of personal or collective value. To recognise this dilemma is to recognise wellbeing as a field of power (p. 38).

A key juncture in the trajectory of neoliberalism was the 2008 Global Financial Crisis (GFC). In response, then-French President, Nicolas Sarkozy commissioned a report on the effectiveness of using Gross Domestic Product (GDP) as a measure of a country's economic performance and social progress (Stiglitz et al., 2009). The report highlighted the limitations of GDP as a valid, reliable predictor of an economy and the health of those living and working within it. Amongst the recommendations were the inclusion of additional indicators beyond GDP with an emphasis on shifting the current measurement system “from measuring economic production to measuring people's wellbeing” (Stiglitz et al., 2009, p. 12). Subsequently, the visibility of wellbeing has grown significantly as states and non-governmental organizations, including the WHO and OECD, adopt new models and frameworks to re-balance economic and social priorities.

Notably, there are a number of states that have introduced national wellbeing frameworks including: Bhutan's Happiness index, the Welsh Wellbeing of Future Generations Act, Sweden's New Measures for Prosperity, and New Zealand's Living Standards Framework which includes a “wellbeing budget”. At this point we briefly focus on New Zealand as it is not only one of the world's first “neoliberal nations”, it has also been one of the most explicit and comprehensive in adopting wellbeing into its state architecture. Consistent with international approaches, the New Zealand wellbeing model is based on aggregated individual dashboard indicators, underpinned by capital investment in areas that are designed to secure future wellbeing (Treasury, 2018). Driven by subsequent “wellbeing budgets” (2019–2023), such measures are now ubiquitous in the strategies and programmes of all state agencies. Yet, for all its purported and perceived benefits the wellbeing budget has done little to address wealth inequality, homelessness, employment insecurity and labor exploitation. Nor has it improved levels of individual and collective health and wellbeing. On the contrary, by any standard quantitative or qualitative measure, society's overall economic, health and social wellbeing has declined (McClure, 2021). Moreover, underscoring the entire discourse of wellbeing is the highly contested axiom that any state and institutional problems can be redefined and reassigned as individual challenges and responsibilities (Rose, 1999). According to Sointu (2005, p. 255–256): “Whereas wellbeing appears to have been an issue pertaining to the “body politic” in the mid-1980s, it now appears to have become a question almost solely related to the context of the “body personal”.”

Consider the status of wellbeing in the workplace. Following the trend within the public sector, the private sector's concern for “how to look after one's self for work” has resulted in an industry of consultants/coaches/specialists that provide wellbeing services (Cederström and Spicer, 2015). As a result, we are witnessing the emergence of “high performance workplace programs” where “wellbeing champions” act as healthy role models for others to follow. The rationale behind these programmes is that staff who are actively managing their wellbeing are more productive, take less sick leave and therefore reduce the burden on their employer. Conversely, the employer is credited with looking after individual employees through funding wellbeing programmes and adding them to their business continuity plans to counter any unforeseen turbulence. This neoliberal transformation of wellbeing has had at least two major and interrelated effects. First, wellbeing now serves as a “policy paradigm by which mind and body can be assessed as economic resources” (Davies, 2011, p. 65). Second, like health, wellbeing has become such a firmly established ideology in society that “failure to conform becomes a stigma” (Cederström and Spicer, 2015, p. 4).

The discussion thus far offers a fairly stern critique of the limits of scholarly conceptualisations of wellbeing and its strategic utilization by state, corporate and other entities. Given the rising global crisis regarding health, there are increasing questions about the theoretical and practical value of instruments such as the WHO-5 index, and the OECD and other frameworks are coming under increasing scrutiny with some authors going so far as to question whether the concept of wellbeing itself is actually counterproductive or even dangerous (Whitaker, 2010; Gruber et al., 2011; Timimi, 2020). Given the complex and contradictory nature of wellbeing along with its enduring, yet precarious, position within policies and programmes, we assert that it may be time to question and disrupt the current hegemony of the concept. Our concerns echo those raised by Cederström and Spicer (2015, p. 11) in relation to wellness:

the pervasive visibility of wellness as a societal mission is having two dominant effects: one, “wellness” has become an ideological normativity which pathologizes those who do not conform to the ideal of wellness or partake of a lifestyle that merits such a label, and two, the relentless pressure to perform wellness might be self-defeating and work against itself in a sense that it could lead to a more alienated, and an unwell society.

In sum, there are numerous limitations associated with current conceptualisations of wellbeing and there may be potential risks associated with its ascendancy as a neoliberal policy instrument that may actually threaten rather than enhance individual and collective health. As such, we propose a potential new research agenda.

## Wellbeing: A proposed research agenda

As a starting point, we propose three broad areas for future research which include wellbeing: (a) as a wicked problem; (b) as part of the process of “wellbeing washing”; and, (c) in relation to alternative futures. We acknowledge that this multi-faceted agenda is not exhaustive, but in combination, these broad lines of research may provide valuable insights in several ways. First, they may help us understand how and why wellbeing is so vexing to define and operationalise within both research and policy. Second, they may alert us to the limits and risks associated with corporate and state (mis)uses of wellbeing as both a commodity and an instrument to monitor and regulate citizens. And, finally, an alternative futures perspective may offer entirely new ways of thinking about health and wellbeing by disrupting existing ontologies and epistemologies.

### Wellbeing as a wicked problem

One established approach to elucidate the contested terrain of wellbeing is to examine it as a “wicked problem” (Rittel and Webber, 1973; Head, 2019). Generally, wicked problems are “vicious” or “tricky”; that is, they are not easily remedied because of disagreements over how they should be defined and because attempts to “solve” them result in new issues/uncertainties (Sam, 2009; Peters, 2017). In this vein, a wellbeing “deficit” is wicked, owing to the problem's ambiguity, multi-causality and the difficulties in assessing and measuring it (Blackman et al., 2006; Bache and Reardon, 2016; Bache et al., 2016). More fundamentally, *ill-being* raises persistent questions around who should “own” the problem (government, employers, labor unions) and/or why we would expect success/failure from one group or the other. That policies around wellbeing will invariably “fail” thus introduces additional wickedness for planners in the form of political risk (cf. Rittel and Webber, 1973; Lynn et al., 1986). Indeed, what makes wellbeing “tricky” is that any attempt to address it will likely change the problem and create new/unintended ones along the way. As policy initiatives grow for instance, they are likely to spur new “audit regimes” with ever more indicators and benchmarks (that “hit the mark” but “miss the point”)? Will deliberations among field/discipline experts and monitoring units (to establish validity and “good” performance), result in even *more* rigid monitoring around wellbeing? That such neoliberal performance management/measurement tools may well undermine the capacity of non-state actors to deliver wellbeing services, is a paradox unlikely to be resolved any time soon.

A view of wicked problems offers a valuable vantage point because the framework abandons any linear/technocratic view of problem solving; as such it helps direct attention to organizational complexity, the interaction of opposing stakeholders, and the (political) limits of rational planning. In this way, it casts a broad analytical net for understanding the built-in constraints to addressing wellbeing, such as the power of Government Treasury departments to define wellbeing as inputs/outputs, or the capacity for organizations to cooperate on a goal that may be secondary to their core purposes. Secondly, and owing to the issue of complexity, contemporary views on wicked problems tend to advance views on how they should be dealt with e.g., through collaborative networks, partnerships and public participation (Head and Alford, 2015). When applied to wellbeing, these processes merit further analysis for the simple reason that they are likely to be a key site and “contested terrain” for the problem's continual reformulation.

### Wellbeing washing

“Wellbeing washing” derives from similar concepts such as greenwashing, rainbow washing and sportswashing. Each of these concepts represent a strategic attempt to use language and visual imagery as part of an organization's branding and promotional culture to connote something positive, or to minimize and manage reputational risk. Moreover, beyond signifying positive sentiments, concepts like “green”, “rainbow” and “wellbeing” enable organizations to appear virtuous given that the meanings of the words are broad and all-encompassing; flexible with respect to interpretation, manipulation and implementation; and applicable to both individuals and institutions. Arguably, the real power and influence lies primarily in the positive meaning associated with each concept, which results in a halo effect. Thus, even though there is nothing inherently, naturally or essentially good about “wellbeing”, anything associated with it tends to inherit its positive qualities thereby making it a powerful and strategic, albeit mythical, concept and tool that can be used by a range of social actors. Here we can draw a parallel with Coakley's (2015) concept of the “Great Sport Myth” (GSM), which assumes that (1) sport is good and pure; (2) sport's purity and goodness are automatically transferred to those who participate in and/or consume it; and, (3) sport always contributes to individual and community development. Similarly, we might refer to the Great Wellbeing Myth (GWM), whereby the assumed inherent positive attributes linked to wellbeing are inevitably transferred to those individuals, groups, institutions and even states that embrace and implement them. Thus, we should not be surprised that supranational agencies (UN, WHO etc.), corporations and myriad organizations use wellbeing as a virtue-signaling term to launder or “wash” the real effects of some of their objectives and practices. This is often achieved through the use of carefully crafted narratives and images via their public relations agencies and wider promotional culture (Wernick, 1991).

Here, we call for a major line of research that explores the phenomenon of “wellbeing washing” within supranational, nation-state and corporate sectors. Key research questions could include:

- How do supranational, state and corporate actors engage in wellbeing washing, that is, what strategies and narratives are used in their public relations and social responsibility promotional campaigns?
- To what extent do discourses and policies of supranational, state and corporate actors advance a neoliberal agenda that ultimately shifts responsibility for collective problems to individuals?
- What are the effects and consequences (intended and/or unintended) of wellbeing washing promotional campaigns, policies and programs on the real lives of citizens?

Collectively, these types of studies have the potential to advance our understanding of the concept of wellbeing and how it is used (and exploited) by particular interests that, even when well-intentioned, may ultimately do more harm than good and leave unchanged a legacy of systemic social and health problems and inequities. In short, they enable us to envision wellbeing as a contested terrain but also as a field of power (White and Blackmore, 2015).

### Alternative futures: Prospects for a “post-wellbeing world”

In response to attempts by states and corporations to cleanse the pernicious (unintended) consequences of their supposedly pure agendas, we offer the notion of “prosperous descent” (Alexander, 2015) as an alternative to rampant and unnecessary consumption (in this case of wellbeing products and services). At the heart of this concept lies the idea of “voluntary simplicity” (Alexander, 2011), or embracing living “low-impact lifestyles … which are nevertheless rich in their nonmaterial dimensions” (Alexander, 2015, p. xii). An alternative research agenda on wellbeing would be guided by the critical assumption that wellbeing is *not* a tangible goal that individuals can achieve by modifying their behavior or consuming wellbeing products or services (e.g. workshops that inform us how to sleep, eat or breathe well). If wellbeing were conceived of as a nonmaterial aspect that cannot be broken down into measurable units, but rather a by-product of living simply and in concert—not conflict—with nature, the need for states, corporations and individuals to measure wellbeing would simply fall away. Conceived as an extension of our humanity—as opposed to a product of our labor—wellbeing becomes a (natural) outcome of who we *are*, rather than something that we must *do* and account for. An emphasis on being rather than doing is central to Indigenous wellbeing frameworks that foreground connectedness to community and country, the importance of land and landscape to identity, cultural expression, kinship, family and Indigenous ways of knowing (Bourke et al., 2018; McIntosh et al., 2021; Yamane and Helm, 2022).

Having conceived of the pursuit of wellbeing as a “wicked problem”, we are all too aware of our complicity in perpetuating its discourse. Instead, relying on the notion of prefiguration, we advocate “building a new world in the shell of the old” (Shantz, 2005). For Boggs (1977, p. 100), prefigurative politics entailed “the embodiment, within the ongoing political practice of a movement, of those forms of social relations, decision-making, culture, and human experience that are the ultimate goal.” More recently, scholars have distilled this aspect of prefigurative politics as “means-ends equivalence” (Maeckelbergh, 2011; Yates, 2015). A prefigurative approach to wellbeing would, therefore, reject the neoliberal idea that being well is a personal responsibility that can be met through additional labor or superfluous consumption. Instead, an alternative wellbeing research agenda would focus on opportunities to establish more meaningful connections with the communities (people) and environments (places) that we belong to, and less on commodities (things) or subjective states of being that supposedly ensure or indicate wellbeing.

According to White and Blackmore (2015, p. 5): “the diversity, volume and velocity in references to wellbeing suggest a cultural tide that sweeps together a range of different interests and agendas”. This essay has outlined the contested terrain of wellbeing by locating it within the context of neoliberalism and the range of supranational, state and corporate interests that use the concept to advance particular interests. To this extent we assert that wellbeing constitutes a “field of power” (White and Blackmore, 2015) and, as such, it is essential that scholars, policy makers and citizens explore “what and whose values are represented, which accounts dominate, what is their impact and on whom” (Scott, 2012, p. 4). We hope our critical assessment, including the proposed agenda for future research, will inspire other scholars to explore.

## Data availability statement

The original contributions presented in the study are included in the article/supplementary material, further inquiries can be directed to the corresponding author/s.

## Author contributions

All authors listed have made a substantial, direct, and intellectual contribution to the work and approved it for publication.

## Conflict of interest

The authors declare that the research was conducted in the absence of any commercial or financial relationships that could be construed as a potential conflict of interest.

## Publisher's note

All claims expressed in this article are solely those of the authors and do not necessarily represent those of their affiliated organizations, or those of the publisher, the editors and the reviewers. Any product that may be evaluated in this article, or claim that may be made by its manufacturer, is not guaranteed or endorsed by the publisher.

## Footnotes

^1^The five statements include: (1) ‘I have felt cheerful and in good spirits', (2) ‘I have felt calm and relaxed', (3) ‘I have felt active and vigorous', (4) ‘I woke up feeling fresh and rested' and (5) ‘My daily life has been filled with things that interest me'. With respect to determining a person's wellbeing, the total raw score, ranging from 0 to 25, is multiplied by 4 to give the final score, with 0 representing the worst imaginable wellbeing and 100 representing the best imaginable well-being.

^2^Global Wellness Institute is a non-profit organization with a mission to empower wellness worldwide by educating the public and private sectors about preventative health and wellness https://globalwellnessinstitute.org/.
